# DeepFRAG: a method for cancer detection based on DNA fragmentomics and deep learning

**DOI:** 10.1093/bioadv/vbag024

**Published:** 2026-01-27

**Authors:** Andrey Koch, Eldar Giladi

**Affiliations:** Laboratory Corporation of America Holdings (Labcorp), Burlington, NC 27215, United States; Laboratory Corporation of America Holdings (Labcorp), Burlington, NC 27215, United States

## Abstract

**Motivation:**

Cancer screening using liquid biopsy technology has become standard in modern clinical and preventive oncology. This method analyzes cell-free DNA (cfDNA) circulating in a patient’s bloodstream. While mutation-based diagnostics using deep exome sequencing are highly sensitive and specific, an alternative approach involves examining cfDNA fragment size distribution profiles. This method is less expensive and can be derived from low-depth whole genome sequencing (WGS).

**Results:**

Our study presents DeepFRAG: a new cancer detection method based on deep learning analysis of cfDNA fragment size distribution profiles using wavelet transform. We utilized two independent cohorts comprising 73 patients with stage III and IV cancers (breast, colorectal, pancreatic, lung, and liver) and 80 healthy individuals. We introduced an original data augmentation technique specific to WGS fragment size data, ensuring sufficient data for training the deep learning model. The proposed method demonstrated high accuracy, with a median test AUROC (area under the receiver operating characteristic curve) of 0.974 and a sensitivity of 96.1% at 98.8% specificity. Our approach offers several advantages, including high accuracy, cost-effectiveness, robustness, and suitability for detecting major cancer types. This method represents a promising advancement in cancer screening technology, expanding the options available for noninvasive cancer detection, with the goal of improving patient outcomes.

**Availability and implementation:**

Data and source code are available at https://github.com/andreykoch/DeepFRAG

## 1 Introduction

### 1.1 Background and motivation

Over the past decade, there has been a significant rise in studies utilizing liquid biopsy technology. These studies typically involve sequencing and variant analysis of the cell-free DNA (cfDNA) circulating in a patient’s bloodstream for applications such as cancer diagnostics (Kwapisz 2017, Zviran *et al*. 2020), organ transplant monitoring (Lo *et al*. 1998), and prenatal screening (Lo *et al*. 1997, Ehrich *et al*. 2011, van der Meij *et al*. 2019). In the context of cancer testing, the primary drawback of this mutation-based approach is that it often requires deep sequencing to detect low-frequency tumor-derived variants, making the assay more costly.

An alternative use of cfDNA leverages distinctive patterns in the distribution of cfDNA fragment sizes (FS) to detect different sources of DNA (e.g. DNA derived from healthy versus tumor cells)—a field known as “fragmentomics.” Though fragmentomics often encompasses additional cfDNA features (such as jagged ends and end motifs), this paper focuses specifically on FS distribution profiles.

Whole genome sequencing (WGS) is capable of detecting DNA fragments up to 1000 base pairs (bp) (Thierry 2023) and allows for the construction of FS probability distribution profiles. Differences in FS distributions between healthy and cancer populations form the foundation for cancer detection. A key advantage of using cfDNA FS information for cancer prediction is that FS profiles can often be derived with low sequencing depth (as low as 0.1×), making it a cost-effective approach. In addition to its applications in cancer prediction (Ivanov *et al*. 2015, Qi *et al*. 2023, Thierry 2023), FS distributions may also aid in the identification of a cancer’s site of origin (Snyder *et al*. 2016, Cristiano *et al*. 2019) and offer other diagnostic applications (Lo *et al*. 2021).

**Figure 1:**
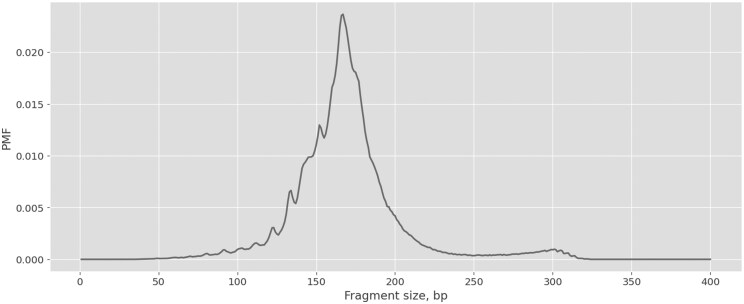
Typical FS PMF profile in the range from 1 to 400 base pairs.

A typical FS profile in a healthy individual has a multimodal structure, with an oscillatory region with a 10-base pair (bp) periodicity (an effect attributed to the wrapping of DNA around histones within the cell nucleus; Qi *et al*. 2023, Thierry 2023), and a prominent peak around 166 bp (Fig. 1). In the context of cancer, cfDNA fragments derived from tumor cells tend to show a higher density of shorter cfDNA fragments compared to the FS profile derived from healthy cells; these size differences are expected to arise from changes in nucleosome packing, resulting in differential degradation of the DNA by nucleases (Thierry 2023). Most current approaches to cancer prediction from cfDNA FS distributions rely on detecting differences in the abundance of shorter versus longer fragments between healthy and cancer samples (e.g. Mouliere *et al*. 2018, Cristiano *et al*. 2019). We hypothesize that most existing cancer detection methods underutilize the rich structural information embedded within FS profiles.

### 1.2 Our approach

Wavelet transform analysis offers a powerful form of spectral analysis, translating the original signal into a space-frequency domain. By capturing localization in both space (FS) and frequency, wavelet analysis is well suited for analyzing the complex structure of FS distribution profiles (Burrus *et al*. 1998). Prominent fluctuations in the FS profile typically range from tens to a few hundred bp, resulting in a profile length between 150 and 400 bp (e.g. Fig. 1). Accordingly, the wavelet transform coefficients produce a vector of comparable length. A recent study demonstrated the power of applying wavelet transforms, as well as Fourier transforms, to cfDNA fragment length distributions to predict tumor content in liquid biopsies from cancer patients (Cardner *et al*. 2023).

Machine learning (ML)-based classification relies on effective feature extraction from data—in our case, the wavelet transform coefficients of FS profiles. While heuristic feature engineering could be applied, automated extraction of the most relevant features by a ML model is preferable. Deep learning models are well-suited for high-dimensional data and are expected to perform predictive feature extraction from FS profiles more effectively than traditional approaches. Recent studies in fragmentomics have focused on the use of machine learning (specifically, deep learning models) to garner additional information from the multi-dimensional data of FS profiles. For example, Kim *et al*. (2024) developed a deep learning model that combines cfDNA methylation markers and FS profiles to distinguish lung cancer patients from healthy individuals, while Shen *et al*. (2024) built a deep learning model called EMIT that uses cfDNA end-motifs to discriminate between individuals with and without cancer.

In this study, we proposed a method called “DeepFRAG” for multi-type cancer detection from cfDNA FS profiles that combines wavelet analysis with deep learning to optimize predictive accuracy.

## 2 Materials

### 2.1 Patient dataset construction

The data used for this study were derived from two independent cohorts. First, the “UCSD” cohort, featured 68 stage III and IV cancer (breast, colorectal, pancreatic, lung, liver) patients who underwent treatment at the UCSD Moores Cancer Center along with 20 healthy donors (Jensen *et al*. 2019). Second, the “Commercial” cohort, comprised 14 colorectal stage III cancer patients, who were treated at several American hospitals, and 60 accompanying healthy patients (Discovery Life Sciences 2022). By combining healthy donors from two sources the final size of the healthy cohort reached 80 samples.

For most patients in both cohorts, multiple samples were collected over time based on the treatment plan. Among the UCSD cancer cohort, only pre-treatment samples were included, totaling 68 samples. In the Commercial cancer cohort, all patients underwent surgery, and their cell-free tumor DNA (ctDNA) levels were measured pre-surgery and 7–11 days post-surgery using a highly sensitive tumor-informed SNV-based method (limit of detection: 0.005%) as described by Rubio-Alarcón *et al*. (2025). From this cohort, 14 samples included five pre-surgery cases with ctDNA levels exceeding 10%, which were added to the cancer cohort, bringing its total to 73 patients (Table 1).

**Table 1:** Data amount and splitting between training and testing subsets for original samples and for subsamples.

Data set	Healthy	Cancer	Total
Total set	80	73	153
Training set	60	55	115
Testing set	20	18	38
Total set, bin sampling	360	363	723
Training set, bin sampling	274	268–270	542–544
Testing set, bin sampling	86	93–95	179–181

### 2.2 Patient sample processing

Whole blood was collected from all patients in the amount of ∼10 ml, and cfDNA was extracted from the separated plasma (Ellison *et al*. 2016). The cfDNA quantification, sequencing libraries preparation, and next-generation WGS of all the samples are described in detail in Jensen *et al*. (2019) and Rubio-Alarcón *et al*. (2025). The sequencers used were Illumina’s HiSeq2500^®^ and NovaSeq6000^®^ instruments. Sequencing depth varied from flowcell to flowcell giving genomic coverage in the range from 0.1× to 30×. Sequencing reads in FASTQ files were quality trimmed and aligned to the hg19 human genome (Green 2001) with Bowtie2 (Langmead *et al*. 2019) and BWA-MEM2 (Vasimuddin *et al*. 2019) aligners. Paired-end sequencing allowed quantification of DNA FS, and the pybam Python package (Longinotto 2017) was used to process BAM files and aggregate fragments in equal-sized (1M bp) non-overlapping segments spanning the whole autosome.

## 3 Methods

### 3.1 Fragment size profiles

For all downstream analysis, for a given sample, we aggregated DNA fragments in the [51, 250] range, where most FS variability is observed, and computed probability mass function (PMF) profiles [also called interchangeably “fragment size (FS) profiles”]. For a given genomic region, PMF of a fragment size l is defined as $\mathrm{PMF}(l)=N_{l}/\sum_{j=k}^{m}N_{j}$, where $N_{l}$ is the number of fragments of size l whose start coordinates fall in that region, and (k, m) is the fragments size range of consideration so that $\sum_{j=k}^{m}\mathrm{PMF}(j)=1$.

### 3.2 Baseline model

To establish baseline performance for our method, we utilized the ratio of FS PMF profiles, integrated over two size intervals: shorter fragments [(100–150) bp] and longer fragments [(151–220) bp], capitalizing on the fact of shorter cfDNA fragments prevalence in cancer samples, as proposed by Cristiano *et al*. (2019). Additionally, we used two more engineered features that proved informative from previous (unpublished) results: average value of FS corresponding to the part of PMF curve above 80^th^ percentile; and the area under the PMF curve right of the theoretical PMF peak at 166 bp. The latter reflects skewness, while the former captures shift in the peak of FS mass distribution in cancer patients. These three benchmark features served as predictors in the baseline model.

We allocated 75% of the dataset for training ML classifiers using logistic regression (LR) and random forest (RF) algorithms with benchmark features (Table 1). The remaining 25% was reserved as a held-out test set to evaluate performance metrics, including accuracy, specificity, sensitivity, and the area under the receiver operating characteristic curve (AUROC). Additionally, we reported area under the precision-recall curve (AUPRC) due to a moderate class imbalance in the data.

To mitigate potential bias from random splits, we performed the train–test splitting procedure 20 times. The median values of performance metrics across these iterations were reported to ensure robustness. Both ML models were implemented in Python 3 using the scikit-learn library (Pedregosa *et al*. 2011).

### 3.3 Discrete wavelet transform of fragment size profiles

As previously noted, a typical cfDNA FS profile exhibits a multi-modal pattern, with peaks at various scales and a prominent peak around 166 bp (Fig. 1). In the 50 to 150 bp range, the profile displays an oscillatory pattern with approximately 10 bp periodicity.

The Fourier transform of a time signal provides frequency localization but lacks time localization. Specifically, each Fourier transform coefficient represents the signal’s energy at a specific frequency. In contrast, a wavelet transform (Burrus *et al*. 1998) offers localization in both time and frequency domains. Each wavelet transform coefficient reflects the signal’s energy within a particular time and frequency range, with the time range varying across scales. Notably, shorter time ranges correspond to broader frequency ranges. In our context, we can substitute time with FS and apply the wavelet transform to the PMF of the FS profile. The localization property in both FS range and frequency range of wavelet analysis is particularly well suited to the complex nature of the FS PMF. We used the discrete wavelet transform (DWT) implemented in the pywt Python package (Lee *et al*. 2019).

### 3.4 Data augmentation

The concatenation of all wavelet coefficients into a single data vector provides a rich set of potential predictive features. Selecting the most relevant features, however, poses a challenge. Advanced methods such as deep learning and tree-based approaches—utilizing unit learners like neurons in neural networks and decision stumps in tree-based models—are well-suited to handle the multidimensional complexity of this input data. In this study, we developed a deep neural network (DNN) to analyze FS profiles, leveraging its demonstrated success across the range of domains including healthcare (LeCun *et al*. 2015, Miotto *et al*. 2016, Hannun *et al*. 2019).

A primary challenge in implementing a DNN is the requirement for a large training dataset, typically consisting of at least several hundred examples. Moreover, when the dimensionality of the input vector exceeds the size of the training set, the problem of finding an optimal solution becomes ill-posed—not only for DNNs but for most machine learning methods in general. This situation creates a strong need to expand the training set. When conventional methods of acquiring more data are exhausted, data augmentation becomes a practical solution. Data augmentation involves applying a series of minor, non-significant transformations to the original data vector to generate new data vectors. In image recognition, for example, this is commonly achieved by distorting, cropping, or rotating the original image (e.g. Shijie *et al*. 2017).

In our context, cfDNA fragments distributed across the human genome provide a natural mechanism for data augmentation by sampling fragments from various genomic regions. For our augmentation strategy, we divided the autosome into equal intervals (bins) of 1 million bp and randomly sampled without replacement fragments from those bins to create individual FS PMF profiles (that we call “subsamples”). This approach ensures that fragments are not reused between subsamples, allowing each original sample to produce multiple independent examples that share the same class label. To maintain the smoothness of PMF profiles, each subsample included at least 1 million fragments. If fragment coverage within a bin did not satisfy this threshold, we sampled additional bins until reaching the minimum fragment count. We kept the number of subsamples per patient minimal to avoid multi-collinearity between them and to allow for consistency across patients.

**Figure 2:**
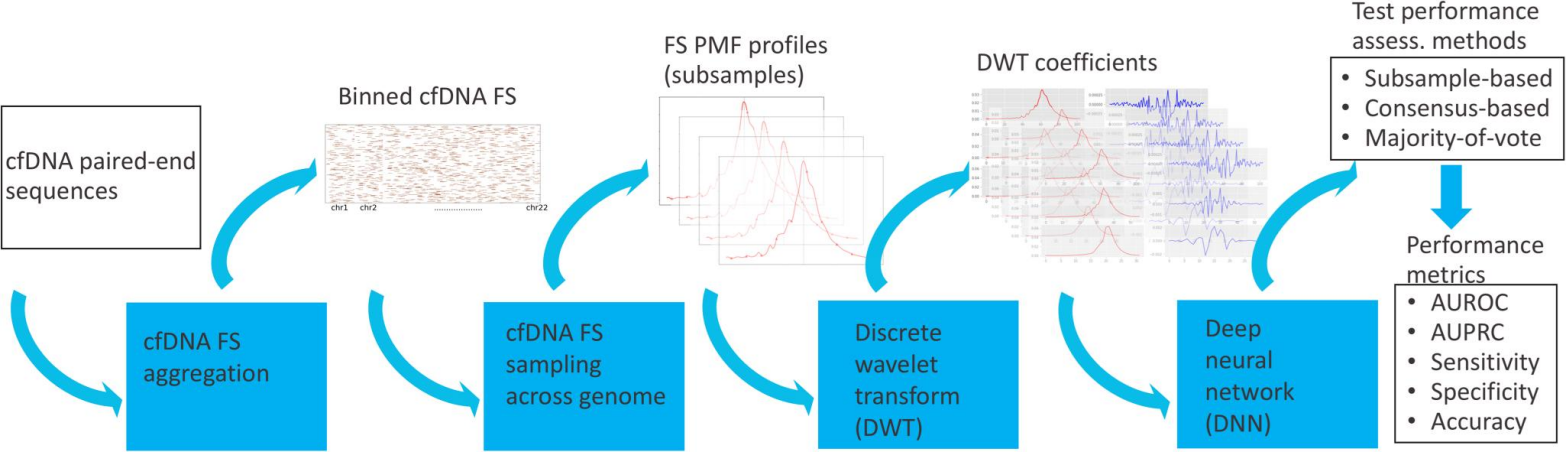
The DeepFRAG method workflow steps for the data [top row: cfDNA sequences; cfDNA fragment sizes (FS); FS probability mass function (PMF) profiles for subsamples; discrete wavelet transform (DWT) coefficients of FS PMF profiles; sample- and patient-level class predictions; model test performance metrics] and computational [bottom row: cfDNA FS aggregation into genomic bins; cfDNA FS sampling; FS PMF computation; computation of DWT coefficients; deep neural network (DNN) training; cancer status predicting; test performance assessment] pipelines.

The main workflow steps of the data and model (computational blocks) pipelines are given in Fig. 2.

### 3.5 Assessment of the test model performance

Assessing the model performance with the current set-up (where multiple samples represent the same patient) can be ambiguous. To address this, various assessment methods were used to determine performance and then compared against each other. The first assessment method was based on treating all subsamples independently with no regard to the patient they represented. This subsample-based method is more intuitive and would be most readily accepted if every subsample represented a single patient. The second method tied subsamples to the original patient and made a final class prediction per patient rather than per subsample. Within this method, two separate performance assessment modes were considered: (i) Consensus vote assessment: the patient class is predicted only if all the subsamples representing a patient agree on its class; if there is no consensus among the subsamples’ predictions, the patient class is predicted as “undefined”—such cases are nevertheless counted toward false positives or false negatives in the test performance computation; and, (ii) Majority-of-vote assessment: the patient class is predicted based on the majority of subsamples belonging to the same patient. The consensus method represents the most conservative approach.

To alleviate a moderate class imbalance in our data set (80 healthy versus 73 cancer samples), the number of cancer subsamples per patient was kept at 5 (except one sample whose lower coverage permitted production of only 3 subsamples), and the number of healthy subsamples equaled either 3 or 5 (and averaged 4.5 over all patients of this class). Maintaining an odd number of subsamples ensured “breaking a tie” in the case of majority-of-vote assessment, with minimum support of all performance metrics for any given patient being 60% (3/5). Additionally, the constraints of multi-collinearity and consistency across patients limited the number of bins because of low coverage in some samples.

### 3.6 Training–testing setup

To minimize bias in the test performance of our model, we employed multiple train–test splits. To avoid data leakage between training and testing subsets, the data splitting was performed on the patient basis such that subsamples belonging to a given patient appeared either in the training subset or in the testing subset, but never in both. Moreover, when sampling DNA fragments from genomic bins, different random seeds were used for different train–test splits, ensuring diverse subsample combinations across splits. To account for the stochastic nature of DNNs, we further retrained the model multiple times within the same data split.

During the data splitting process, we used stratified sampling to maintain a consistent representation of different flowcells across the training and testing datasets. This approach resulted in a well-balanced and enriched dataset of 723 data points, with 543 allocated for training (Table 1). The training methodology for DNN is described in the subsequent section. In addition, we conducted a 10-fold cross-validation (CV) using one of the subsampling datasets that resulted from combining training and testing parts of a fixed data split. Multiple DNN re-trainings were performed within each fold to report median CV performance.

### 3.7 Deep learning model

The feature vector size for our DNN was 224 when DWT coefficients were used as input, and 200 when FS PMF profiles were used. The DNN used is a feed-forward, densely connected network with three hidden layers consisting of 64, 32, and 16 neurons, respectively. All neurons in hidden layers used leaky ReLU activation units, and a dropout normalization was applied to each hidden layer. The DNN used Adam optimizer, and a binary cross-entropy loss function.

The model was trained until convergence was reached, defined by the following criteria: (i) the loss function averaged over the last five training epochs dropped below 0.1; and (ii) stabilization was seen from epoch to epoch (i.e. the range of the loss function over the same period, relative to the mean loss, did not exceed 15%)—Stopping rule 1. In addition, the maximum number of training epochs was set at 400. For comparison with more established strategies, we also used a validation set-based stopping rule. When validation loss (computed on an additional validation set that resulted from splitting 10% off the training set) increased for five consecutive training epochs, the training stopped and the saved model from five epochs ago was used to assess test performance—Stopping rule 2. The DNN model was implemented in Tensorflow with Keras API using Python 3.

## 4 Results

### 4.1 Baseline model

For assessing the test performance of the baseline models, we used the augmented dataset for consistency in comparison with the DNN, even though these models could be trained with the original dataset. By default, for the baseline models, we are reporting in Table 2 test performance metrics computed using the majority-of-vote method. The complete set of metrics computed with all three assessment methods is given in Table S1, available as supplementary data at *Bioinformatics Advances* online. The LR model trained with benchmark features yielded 0.822 median test AUROC with 82.1% accuracy and 78.9% sensitivity at 87.5% specificity (Table 2). The RF model noticeably improved test performance, yielding 0.883 AUROC with 88.5% accuracy, with test sensitivity of 84.2% at 95.0% specificity.

**Table 2:** Median test model performance.

Model	Feature	Perf. assess.	Acc.	Spec.	Sens.	AUROC	AUPRC	Time
LR	Bench.	Majority	82.1	87.5	78.9	0.822	0.762	0.05

RF	Bench.	Majority	88.5	95.0	84.2	0.883	0.84	0.14

		Subsamp.	95.3	95.7	94.7	0.954	0.931	
RF	DWT	Consensus	92.3	90.0	94.7	0.922	0.867	0.29
		Majority	94.9	95.0	94.7	0.949	0.94	

		Subsamp.	95.4	97.2	94.7	0.964	0.961	
DNN	DWT	Consensus	89.7	92.5	90.8	0.897	0.889	15.4
		Majority	97.4	98.8	96.1	0.974	0.95	

		Subsamp.	95.1	96.6	94.2	0.961	0.955	
DNN	FS PMF	Consensus	91.0	95.0	89.5	0.911	0.893	32.98
		Majority	94.9	95.0	96.1	0.949	0.941	

Median test model performance [accuracy, specificity, sensitivity (in %), AUROC, AUPRC] over 20 test sets assessed using three methods (subsample-based, consensus-based, and majority-of-vote) for different classifiers (LR—logistic regression; RF—random forest; DNN—deep neural network) and predictive features (Bench.—the three benchmark features; FS PMF—fragment size probability mass function profile; DWT—discrete wavelet coefficients of FS PMF profile), along with training time (in sec.) for a single training session.

### 4.2 Deep learning model with discrete wavelet transform coefficients

#### 4.2.1 Comparison of performance assessment methods

**Figure 3:**
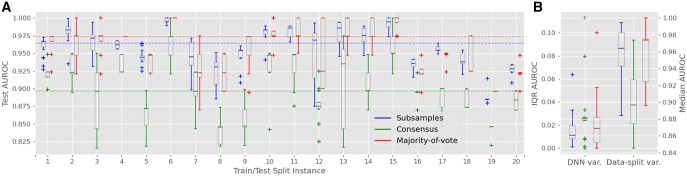
Test AUROC results for the deep learning classifier trained with DWT coefficients. (A) Boxplots for 20 random train–test splits. Each boxplot characterizes variability over 20 model re-trainings within a fixed data split. Results for three performance assessment methods (in the groups of three boxplots) are shown: subsample-based (left), consensus-based (center), and majority-of-vote (right). Dashed lines mark the overall median values: 0.964 for subsample-based method, 0.897 for consensus, and 0.974 for majority-of-vote (Table 2). (B) Boxplots of interquartile ranges [IQR, from (A)] summarizing variability due to DNN behavior (left side); boxplots of medians [from (A)] summarizing variability due to data composition (right side).

We utilized a DNN model and evaluated its performance through multiple re-runs as outlined in the design. The experiments conducted showed that on average, the consensus-based method reports performance 5%–8% lower than the majority-of-vote method (e.g. Fig. 3b; Table 2). When comparing majority-of-vote with the subsample-based method, for different data splitting instances the former can report higher performance than the latter and vice versa (e.g. Fig. 3a). On average, however, the majority-of-vote method gives slightly higher (∼1%) performance (e.g. Fig. 3b; Table 2). Despite reporting model test performance given by all three methods, we give preference to the patient-based methods (majority-of-vote and consensus) due to the patient-based prediction setting.

#### 4.2.2 Deep neural network stopping strategies

Figure S1 presents a comparison of the 10-fold CV performance for the DNN trained using the two previously described stopping rules. Stopping rule 1 consistently yields higher median AUROC values across all three performance methods, indicating superior predictive performance. It also demonstrates greater robustness, with AUROC variability as measured by the interquartile range (IQR). Indeed, the IQR of stopping rule 1 is roughly half of the one observed with stopping rule 2. Although stopping rule 2 could potentially be tuned (e.g. by adjusting the number of training epochs to account for the continuous rise in validation loss), we chose to use stopping rule 1 for all DNN experiments due to its stronger CV performance, independence from a validation set, and overall stability.

#### 4.2.3 Test model performance

Figure 3a presents box plots of the test AUROC derived from the three performance assessment methods (i.e. subsample-based, consensus-based, and majority-of-vote) for the DNN trained with DWT coefficients. Each box plot summarizes 20 re-runs of the model for a fixed train–test split, derived from total 20 different train–test splits. These box plots capture the variability caused by stochastic factors in the neural network, such as random weight initialization and neuron selection for dropout. Variability along the x-axis reflects changes in train–test data composition and in FS sampled from different genomic bins.

The median test AUROC across all splits for the majority-of-vote method was 0.974 (Table 2; Fig. 3), representing a 10.3% improvement compared to the best-performing baseline model (RF trained on benchmark features; AUROC of 0.883). The subsampling-based method achieved a slightly lower median AUROC of 0.964, while the consensus-based method reported a median AUROC of 0.897.

A similar trend was observed for other performance metrics. The majority-of-vote method achieved a median test accuracy of 97.4%, a median test sensitivity of 96.1%, a median test specificity of 98.8%, and a median test AUPRC of 0.95 (Table 2; Figs S2–S5 respectively). The variability in test AUROC caused by DNN stochasticity, measured as the median of IQR for each data split (Fig. 3b, left), was lowest for the consensus method, at 0.003, second largest for the subsampling method (0.012) and highest for the majority-of-vote method (0.022). In comparison, the variability due to changes in data composition, measured as the IQR of medians across data splits (Fig. 3b, right), was approximately three times greater than the variability from DNN stochasticity. This variability remains similar across all performance assessment methods: 0.04 for subsampling, and 0.051 for majority-of-vote and consensus methods.

#### 4.2.4 Model evaluation: cross-validation versus multiple train–test splitting

As an alternative approach to evaluating the model’s predictive performance, 10-fold CV was used. Its results were in agreement with the findings obtained from multiple train–test splits. Namely, using the majority-of-vote method, the model achieved a median CV accuracy of 98.4%, median CV sensitivity of 100% at 96.6% specificity. Additionally, the median CV AUROC was 0.984, and the median CV AUPRC was 0.972 (see Table S2 and Figs S6–S10).

It is important to note that each train–test split involved a unique combination of subsamples for training and testing, whereas in the n-fold CV setting, the same subsamples were reused across folds for training and validation. This distinction contributed to the greater robustness of the train–test split evaluation method, which lead to its adoption as the primary framework in this study, even though CV results were slightly more favorable.

### 4.3 Random forest model with discrete wavelet transform coefficients

**Figure 4:**
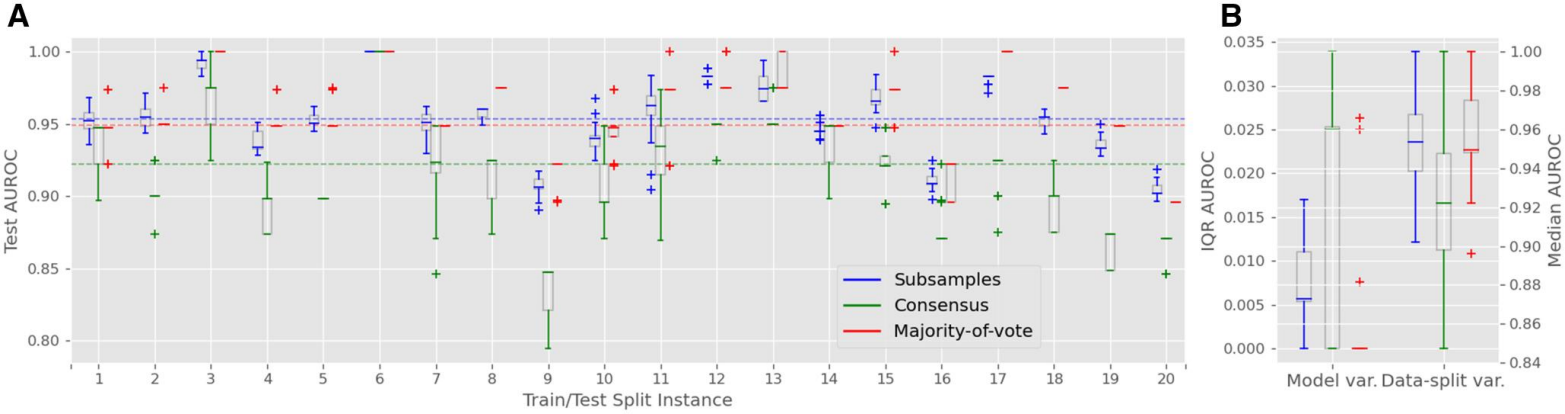
Test AUROC results for the RF classifier trained with DWT coefficients. For figure details refer to Fig. 3 and for the overall median values refer to Table 2.

The achieved AUROC and other metrics represent a significant performance improvement compared to the RF classifier trained with engineered benchmark features. To evaluate the separate contributions of the DWT-based features and the DNN to this performance increase, we trained an RF classifier using DWT coefficients and compared its test performance metrics (Table 2; Fig. 4) with those of the baseline model (Table 2) and the deep learning model (Table 2; Fig. 3).

The RF model trained with DWT coefficients achieved a median test AUROC of 0.949 (assessed by the majority-of-vote method), which is a 7.5% improvement over the baseline model trained with the benchmark features (AUROC of 0.883). It also achieved a median test sensitivity of 94.7% (Table 2; Fig. S12, 95.0% specificity (Table 2; Fig. S13), and median test accuracy of 94.9% (Table 2; Fig. S11). The improvement in RF performance compared to baseline is solely attributed to replacing the benchmark FS-based features with DWT decomposition coefficients of FS profiles.

Further enhancement is achieved using the deep learning classifier, which increases the AUROC by an additional 2.6%. The difference in median test performance between the RF and DNN models with DWT coefficients, as measured by the subsampling method, was not as pronounced as the differences observed with the majority-of-vote method: sensitivity was the same, accuracy changed only by 0.1%, AUROC—by 1%, and only specificity and AUPRC changed by 1.6% and 3.2%, respectively. It is interesting to observe that all three performance assessment methods in RF model trained with DWT setting were aligned much better than in the DNN with DWT setting (Table 2).

### 4.4 Deep learning model with fragment size probability mass function profiles

We also evaluated a model setup where the wavelet transformation (DWT) was omitted, and the DNN was trained directly using FS PMF profiles. Remarkably, the neural network extracted highly comparable predictive information from the PMF profiles as it did with the DWT layer.

**Figure 5:**
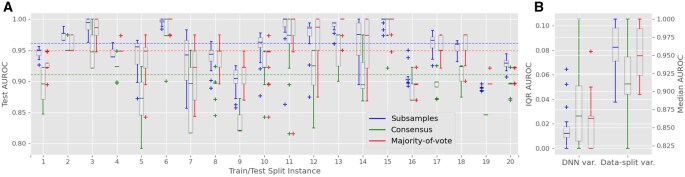
Test AUROC results for the deep learning classifier trained with PMF profiles. For figure details refer to Fig. 3 and for the overall median values refer to Table 2.

For the subsample-based method, all performance metrics were nearly identical between the DNN trained with DWT coefficients and the DNN trained with PMF profiles and varied only in the 0.3%–0.6% range, depending on the metric (Table 2; Fig. 5, Figs S15–S18). The consensus-based method reported even slightly elevated values in DNN with PMF setting, compared to DNN with DWT setting, for all metrics except sensitivity. Using the majority-of-vote method, both setups achieved the same median test sensitivity (96.1%). However, the DNN trained with PMF profiles showed somewhat lower performance in terms of median test AUROC (2.6% lower; Table 2; Fig. 5), median test AUPRC (0.9% lower; Table 2; Fig. S18), median test specificity (3.8% lower; Table 2; Fig. S17), and median test accuracy (2.6% lower; Table 2; Fig. S15) compared to the DNN trained with DWT coefficients. This highly comparable performance is achieved, however, at the cost of doubling the training time when using FS PMF profiles (Table 2).

### 4.5 Additional modeling experiments

Table S1 summarizes the experiments in which the LR model was trained using DWT coefficients, and both LR and RF models were trained using FS PMF profiles. Notably, the LR model yielded measurably inferior performance when trained with either FS profiles or wavelet coefficients, compared to the benchmark feature set. In contrast, the RF model demonstrated improved performance when trained on the full FS probability profile. This performance was almost identical to its performance using wavelet coefficients.

### 4.6 SHAP analysis

To assess feature importance, we conducted Shapley additive explanations (SHAP) analysis (Lundberg and Lee 2017) by sequentially zeroing out five distinct regions of the FS PMF profile: the left oscillatory region, right oscillatory region, mode region, slope region, and tail region. Figure S19 presents AUROC boxplots across 10 CV folds for models trained using DWT coefficients derived from either the full PMF signal or a modified signal with one region removed. The most significant performance decline occurred when either the oscillatory or the mode regions were excluded. This indicates their heightened importance relative to other regions. These areas, which contain pronounced fluctuations in the FS PMF profile, are at the basis of our benchmark features. Consequently, the SHAP analysis validated the use of these benchmark features in our baseline models.

## 5 Discussion

In this study, DeepFRAG, a cancer detection method, was developed. A deep learning model (referred to as the “DNN”) was used to extract predictive features from discrete wavelet transform (DWT) coefficients of cfDNA FS profiles in individuals with and without cancer. The performance of this model was assessed using a subsample-based method, a consensus method, and a majority-of-vote method. The DNN trained with DWT coefficients showed performance (i.e. sensitivity, specificity, AUROC, AUPRC, and accuracy) superior to a RF model trained with benchmark features, as well as a RF model trained with DWT coefficients. The DNN trained with DWT coefficients showed similar performance to the DNN trained with the FS PMF (the standard FS profile), but with a considerably shorter training time.

The decomposition of DNA FS probability profiles using discrete wavelet transform has proven to be an effective method for transforming the original data into a new feature space suitable for machine learning applications in cancer prediction. As compared to the Fourier transform, in which a time signal provides frequency localization, but lacks time localization, the DWT coefficient reflects the signal’s energy within a particular time and frequency range. In this study, we substituted time with FS to apply the wavelet transform to the PMF of the FS profile.

A DNN was chosen to analyze FS profiles in this study because of its ability to handle input data with multidimensional complexity, and because of its demonstrated success across numerous domains, including healthcare (LeCun *et al*. 2015, Miotto *et al*. 2016, Hannun *et al*. 2019). A major challenge in implementing a DNN is the need for a substantial amount of training data. By sampling fragments from individual segments across the whole genome, a new augmented and balanced dataset was created, enhancing the training process for both deep learning and other machine learning models that benefit from this enriched feature space. The DNN in this study was a feed-forward network. Prior studies have shown that feed-forward neural networks with similar architecture proved to perform very efficiently for binary classification problems with 1D feature vectors as input across different domains (e.g. Cloud *et al*. 2019, Jeong and Lee 2021).

The new data augmentation approach presented in this paper introduced some ambiguity in evaluating model test performance. The majority-of-vote method for class prediction, used in this study, is not the most widely adopted but is supported by its robustness. This robustness is ensured by (i) the data augmentation design, which uses an odd number of subsamples to avoid ties in class voting, and (ii) restricting the number of subsamples so that a minimum support threshold of 60% is required to assign a class label. The DNN trained on the DWT coefficients in our study achieved a test median AUROC of 0.974 when evaluated using the majority-of-vote method; this represents a 10.3% improvement over a RF model using benchmark features. The enhanced performance is attributed to the data augmentation (i.e. genome-binning) approach that was employed to solve the data scarcity problem in training deep learning models.

An alternative assessment method, which treated each subsample independently, demonstrated similarly high performance to the majority-of-vote method. This approach is more intuitive and would have been preferred if sufficient data were available, with each subsample corresponding to a single patient. However, within the context of the proposed cancer prediction method, this alternative is not practical.

The most restrictive assessment method, based on consensus among all subsamples to assign a class label, reported a test model AUROC of 0.897, which is 7.9% lower than the majority-of-vote method.

Interestingly, most of the improvement in predictive performance over the baseline model was achieved by introducing new data in the form of DWT coefficients derived from FS PMF profiles, as demonstrated by the performance of the RF model. The application of a deep learning model further enhanced this performance.

It is important to recognize that, as model test performance approaches high levels, achieving even a small increase often demands significantly greater computational resources, increased algorithmic complexity, or both. Consequently, the contribution of deep learning to the success of the proposed algorithm should be considered highly significant.

In addition, it is worth noting that very similar predictive test performance—matching sensitivity, though with somewhat reduced specificity (3.8% lower), accuracy and AUROC (2.6% lower), and AUPRC (0.9% lower)—can be achieved when a deep learning model is trained directly on FS probability profiles. However, without applying the discrete wavelet transform to the FS profiles first, the learning algorithm requires twice as much time to identify a mapping function with comparable predictive power. The considerable training time reduction, together with somewhat better AUROC, AUPRC, specificity, and accuracy, justifies the inclusion of wavelet transformation step in data processing prior to training the deep learning model (Of note, the wavelet transformation step adds a negligible amount of time to data processing, when compared to the time saved in training).

This study contributes to the growing body of research on cfDNA fragmentomics for cancer prediction. In their 2023 publication, Cardner *et al*. introduced a method for estimating tumor content using Fourier and wavelet transform coefficients derived from cfDNA fragment length distributions. These coefficients were used within a beta regression framework. While both approaches utilize wavelet transforms of FS profiles, the key distinction lies in their objectives: Cardner *et al*. focused on quantifying tumor content, whereas our study aimed to classify samples as either disease or healthy. LR can be seen as a special case of beta regression when the response is binary, and the former performed least effectively in our experiments. Unlike beta regression, more advanced models such as RFs and DNNs are better equipped to capture the intricate structure of FS PMF and DWT profiles. Nonetheless, adapting our method to predict tumor content represents a promising direction for future research.

Kim *et al*. (2024) developed a convolutional neural network (CNN) to differentiate lung cancer from healthy patients by combining methylation signatures with cfDNA FS data. Their CNN input was a large 2D table of methylation ratios computed at the intersection of hundreds of 100-bp genomic regions with 10-bp FS intervals. This resulted in a very high-dimensional input vector, which was a potential issue given their small training set of only 144 samples. While we believe the combination of methylation and fragmentomics is a promising approach, Kim *et al*. used FS information only as a coordinate. In our study, we elected to use complex, multi-spectral information from FS distributions for disease detection.

In another study, Shen *et al*. (2024) pre-trained a transformer-based neural network using unlabeled cfDNA end-motif sequences to learn feature representations; a linear classifier was then trained on these representations using labeled cancer/healthy samples to evaluate its diagnostic performance. The end-motif information is another promising biomarker, yet not as complex as FS distribution profiles used in our study. End-motifs can be leveraged as an additional predictive feature in combination with FS distribution in future research.

In summary, DeepFRAG, a method for cancer prediction based on FS profiles proposed in this study, is inexpensive, highly accurate, and robust. A logical next step in this research will be to explore the limits of ctDNA detection using this approach.

## Supplementary Material

vbag024_Supplementary_Data

## Data Availability

The data and source code underlying this article are available at https://github.com/andreykoch/DeepFRAG.
