# Supplementary material for: DeepFRAG: a method for cancer detection based on DNA fragmentomics and deep learning
Source: Bioinform Adv. 2026 Jan 27;6(1):vbag024. doi: 10.1093/bioadv/vbag024 (PMC12973171; doi:10.1093/bioadv/vbag024)
Supplement: vbag024_Supplementary_Data [file vbag024_supplementary_data.docx]

Supplemental Materials to “DeepFRAG: A Method for Cancer Detection Based on DNA Fragmentomics and Deep Learning” by Andrey Koch and Eldar Giladi

| **Model** | **Feature** | **Perf. assess.** | **Accuracy, %** | **Specificity, %** | **Sensitivity, %** | **AUROC** | **AUPRC** | **Tr. time, s** |
| --- | --- | --- | --- | --- | --- | --- | --- | --- |
| LR | Bench. | Subsamp. | 82.5 | 87.5 | 81.1 | 0.824 | 0.78 | 0.05 |
|  |  | Consensus | 78.2 | 80.0 | 73.7 | 0.783 | 0.71 |  |
|  |  | Majority | 82.1 | 87.5 | 78.9 | 0.822 | 0.762 |  |
| RF | Bench. | Subsamp. | 86.6 | 90.3 | 84.2 | 0.865 | 0.821 | 0.14 |
|  |  | Consensus | 78.8 | 75.0 | 73.7 | 0.79 | 0.707 |  |
|  |  | Majority | 88.5 | 95.0 | 84.2 | 0.883 | 0.84 |  |
| LR | FS PMF | Subsamp. | 73.4 | 100.0 | 49.5 | 0.742 | 0.747 | 0.05 |
|  |  | Consensus | 74.4 | 100.0 | 47.4 | 0.737 | 0.73 |  |
|  |  | Majority | 75.6 | 100.0 | 50.0 | 0.75 | 0.744 |  |
| RF | FS PMF | Subsamp. | 93.4 | 95.7 | 92.6 | 0.935 | 0.913 | 0.26 |
|  |  | Consensus | 88.5 | 90.0 | 89.5 | 0.886 | 0.847 |  |
|  |  | Majority | 94.9 | 95.0 | 94.7 | 0.948 | 0.923 |  |
| LR | DWT | Subsamp. | 73.4 | 100.0 | 49.5 | 0.742 | 0.747 | 0.05 |
|  |  | Consensus | 74.4 | 100.0 | 50.0 | 0.737 | 0.73 |  |
|  |  | Majority | 75.6 | 100.0 | 50.0 | 0.75 | 0.744 |  |

Table 1S. Median test model performance (accuracy, specificity, sensitivity, AUROC, AUPRC) over 20 test sets assessed using three methods (subsample-based, consensus-based, and majority-of-vote) for different classifiers (LR – logistic regression; RF – random forest) and predictive features (Bench. – three benchmark features described in Baseline model section; FS PMF – fragment size probability mass function profile; DWT – discrete wavelet coefficients of FS PMF profile), along with training time for a single training session.

| **Model** | **Feature** | **Perf. assess.** | **Accuracy, %** | **Specificity, %** | **Sensitivity, %** | **AUROC** | **AUPRC** |
| --- | --- | --- | --- | --- | --- | --- | --- |
| DNN | DWT | Subsamp. | 96.4 | 93.3 | 100.0 | 0.98 | 0.962 |
|  |  | Consensus | 93.3 | 87.5 | 100.0 | 0.938 | 0.875 |
|  |  | Majority | 98.4 | 96.9 | 100.0 | 0.984 | 0.972 |

Table 2S. Median test model performance (accuracy, specificity, sensitivity, AUROC, AUPRC) over 10 CV folds assessed using three methods (subsample-based, consensus-based, and majority-of-vote) for deep neural network trained with DWT coefficients of FS PMF profiles.


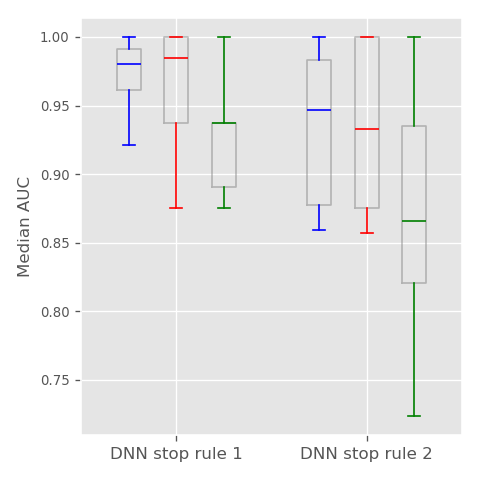


Figure 1S. CV AUROC results for deep neural network trained with DWT coefficients for three performance assessment methods: subsample-based (blue), consensus-based (green), and majority-of-vote (red). Boxplots characterize variability over 10 CV folds, where each data point from which boxplots are drawn represents median AUROC over 20 model re-trainings within a fixed CV fold. Results are shown using models converged with stopping rule 1 (left side) and stopping rule 2 (right side).


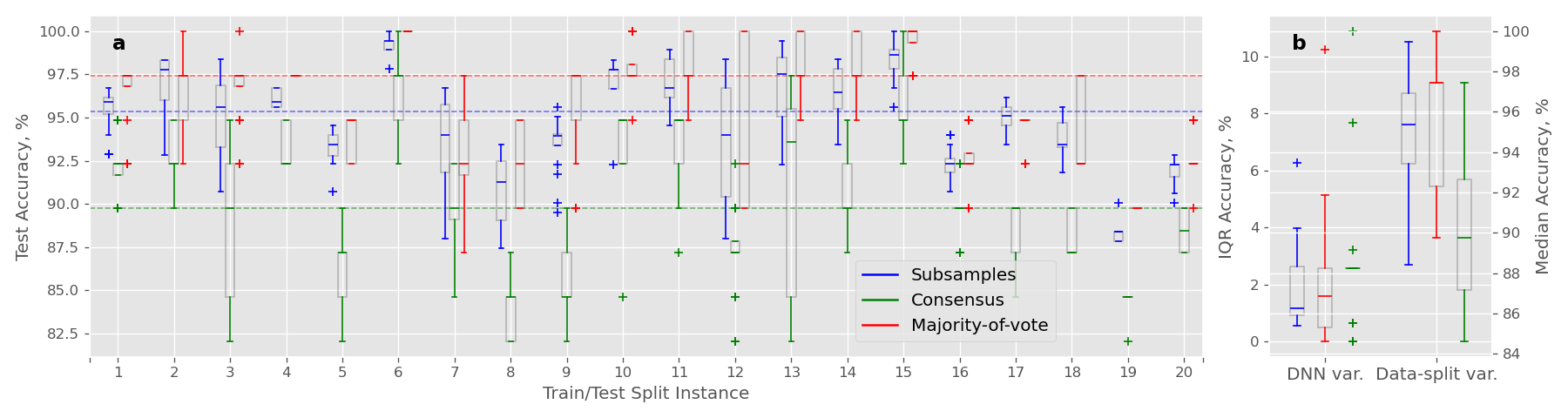
Figure 2S. Test accuracy results for deep neural network trained with DWT coefficients. (a) Boxplots for 20 random train-test splits. Each boxplot characterizes variability over 20 model re-trainings within a fixed data split. Results for three performance assessment methods are shown: subsample-based (blue), consensus-based (green), and majority-of-vote (red). Dashed lines mark the overall median AUROCs: 95.4% for subsample-based method, 89.7% for consensus, and 97.4% for majority-of-vote (Table 2). (b) Boxplots of interquartile ranges (IQR, from (a)) summarizing variability due to DNN behavior (left side); boxplots of medians (from (a)) summarizing variability due to data composition (right side).


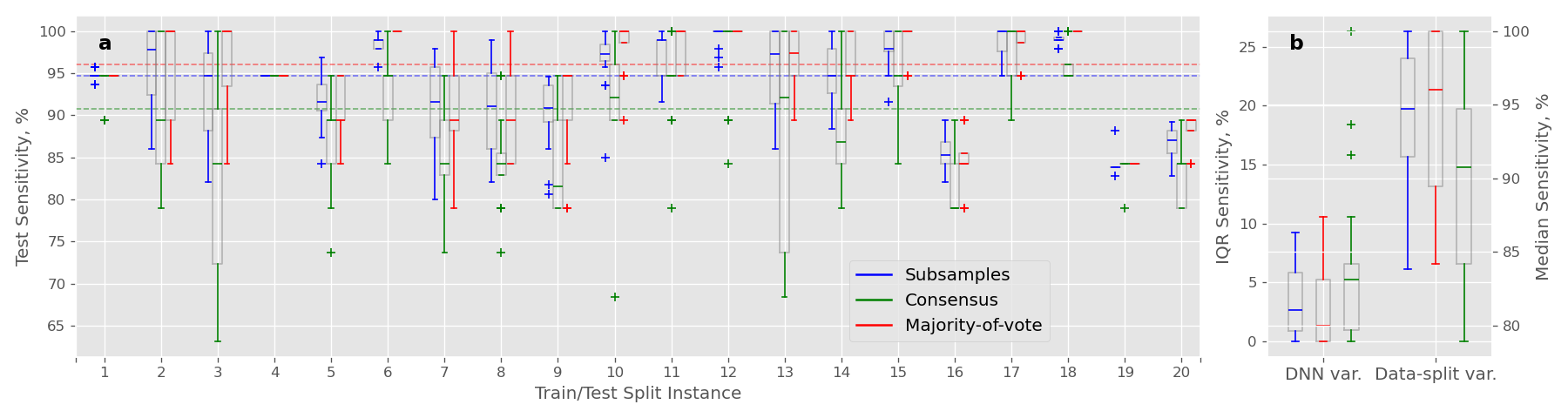
Figure 3S. Test sensitivity results for deep neural network trained with DWT coefficients. For figure details refer to Figure 2S and for the overall median values refer to Table 2.


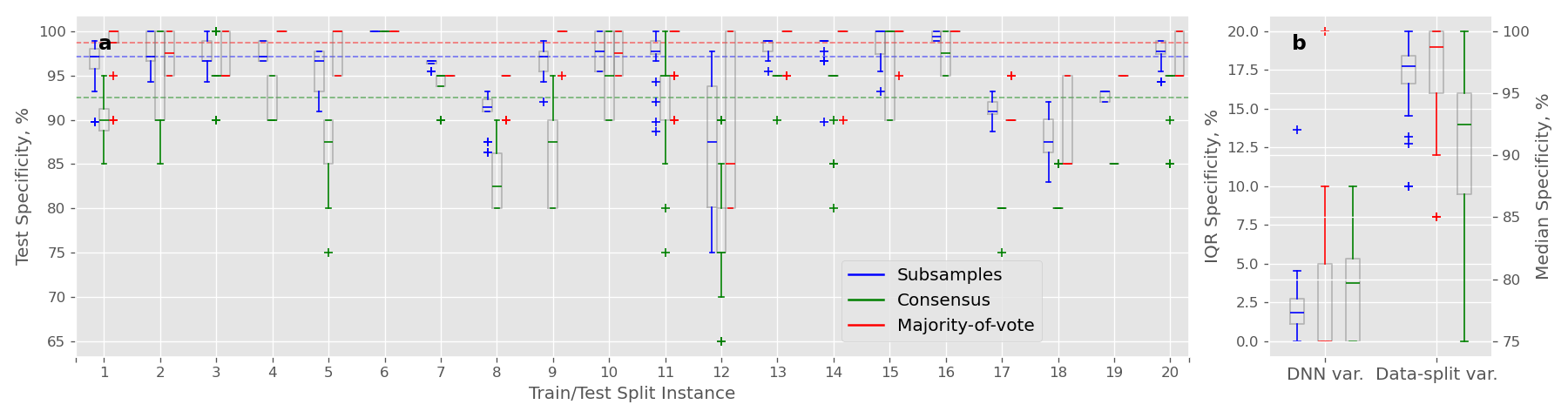
Figure 4S. Test specificity results for deep neural network trained with DWT coefficients. For figure details refer to Figure 2S and for the overall median values refer to Table 2.


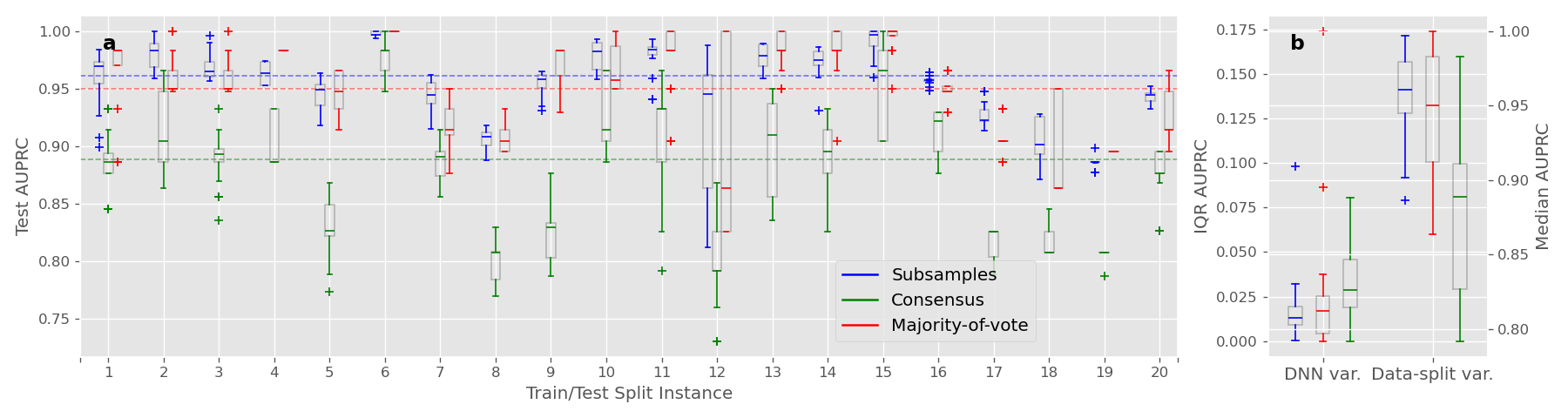
Figure 5S. Test AUPRC results for deep neural network trained with DWT coefficients. For figure details refer to Figure 2S and for the overall median values refer to Table 2.


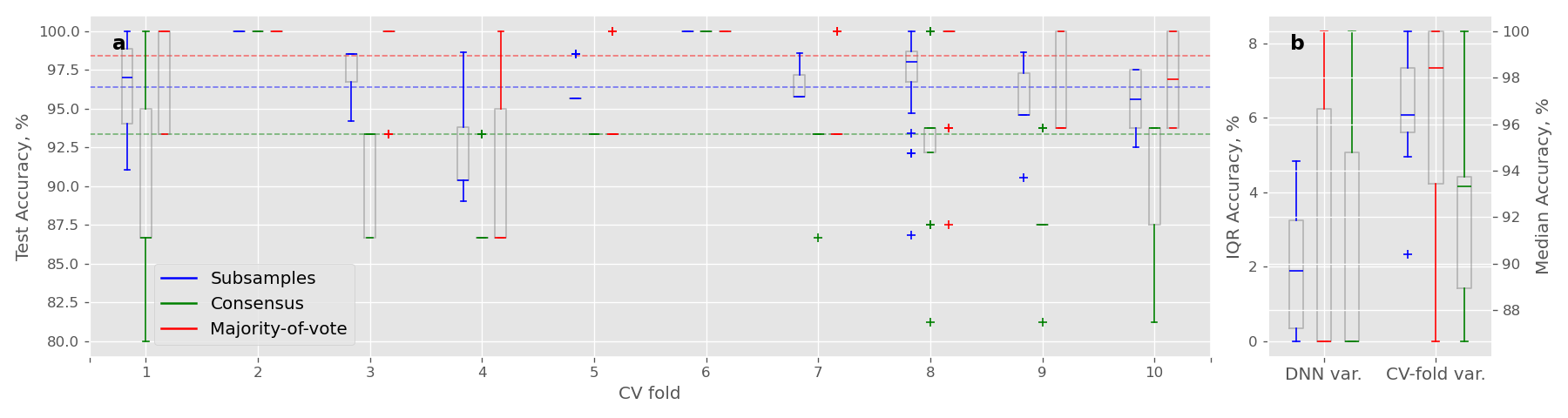
Figure 6S. 10-fold CV accuracy results for deep neural network trained with DWT coefficients. (a) Boxplots for 10 CV folds. Each boxplot characterizes variability over 20 model re-trainings within a fixed fold. Results for three performance assessment methods are shown: subsample-based (blue), consensus-based (green), and majority-of-vote (red). Dashed lines mark the overall median accuracy: 96.2% for subsample-based method, 91.0% for consensus, and 98.4% for majority-of-vote. (b) Boxplots of interquartile ranges (IQR, from (a)) summarizing variability due to DNN behavior (left side); boxplots of medians (from (a)) summarizing variability due to data composition (right side).


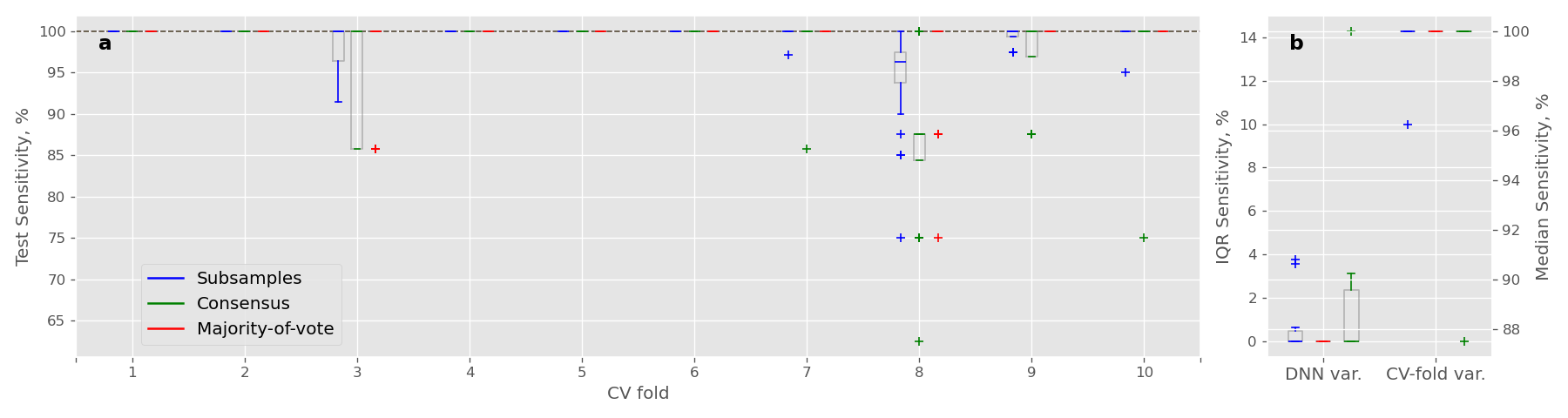
Figure 7S. 10-fold CV sensitivity results for deep neural network trained with DWT coefficients. For figure details refer to Figure 6S and for the overall median values refer to Table 2S.


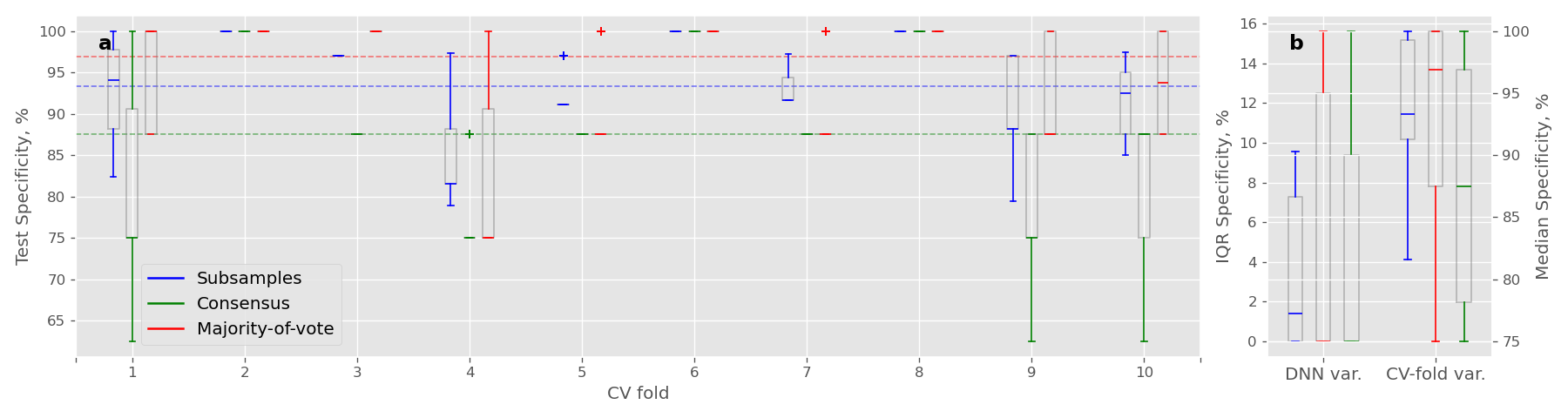
Figure 8S. 10-fold CV specificity results for deep neural network trained with DWT coefficients. For figure details refer to Figure 6S and for the overall median values refer to Table 2S.


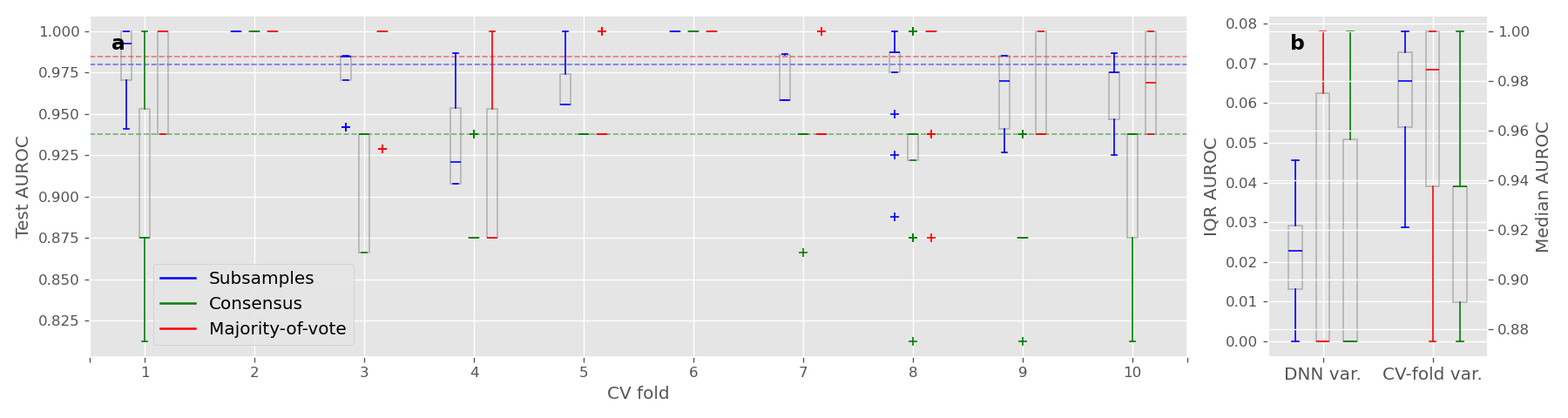
Figure 9S. 10-fold CV AUROC results for deep neural network trained with DWT coefficients. For figure details refer to Figure 6S and for the overall median values refer to Table 2S.


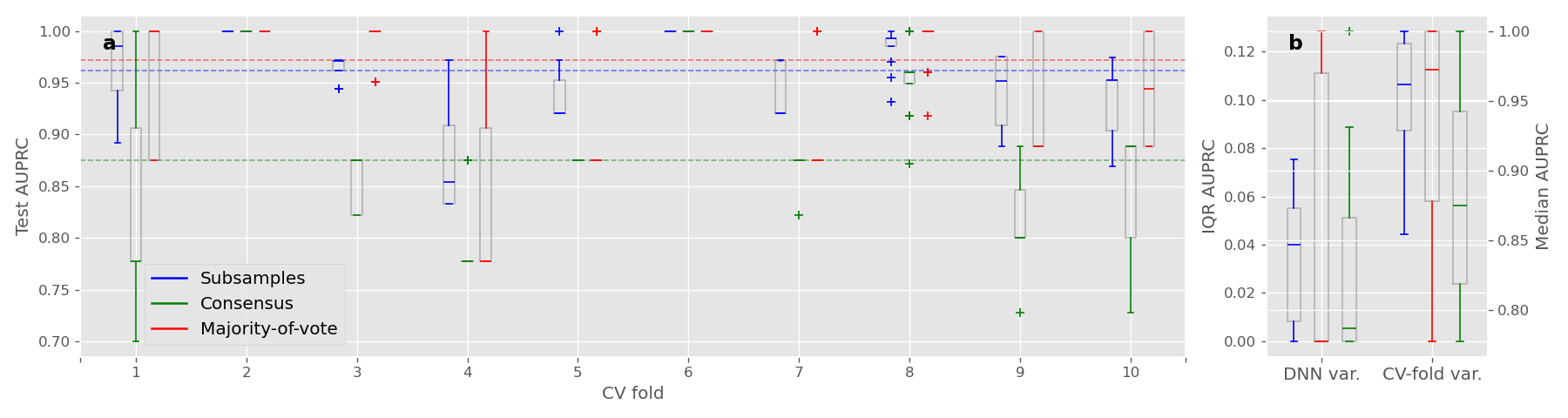
Figure 10S. 10-fold CV AUPRC results for deep neural network trained with DWT coefficients. For figure details refer to Figure 6S and for the overall median values refer to Table 2S.


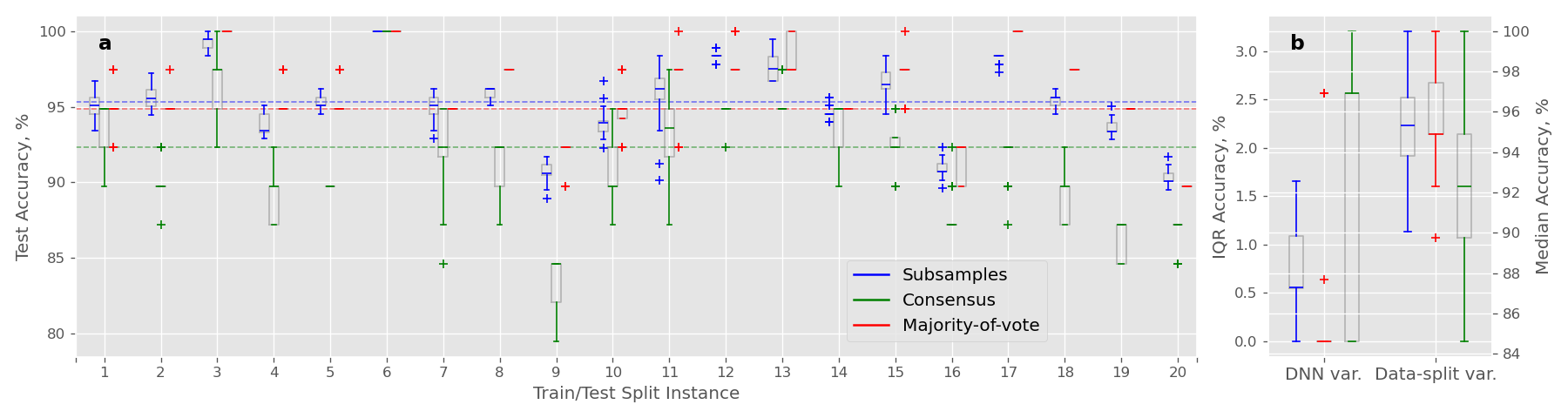
Figure 11S. Test accuracy results for the random forest classifier trained with DWT coefficients. For figure details refer to Figure 2S and for the overall median values refer to Table 2.


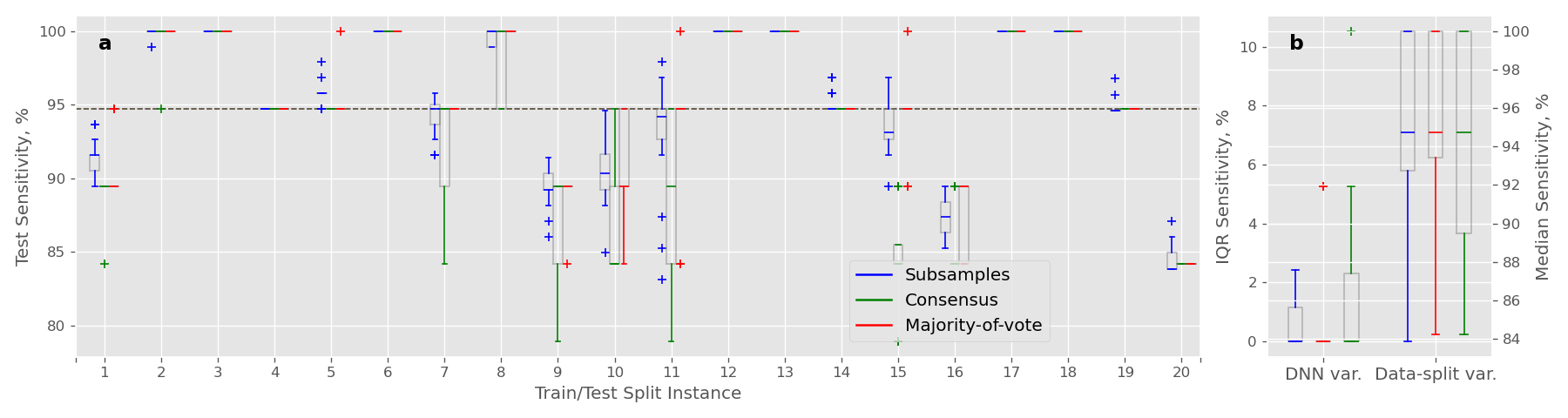
Figure 12S. Test sensitivity results for the random forest classifier trained with DWT coefficients. For figure details refer to Figure 2S and for the overall median values refer to Table 2.


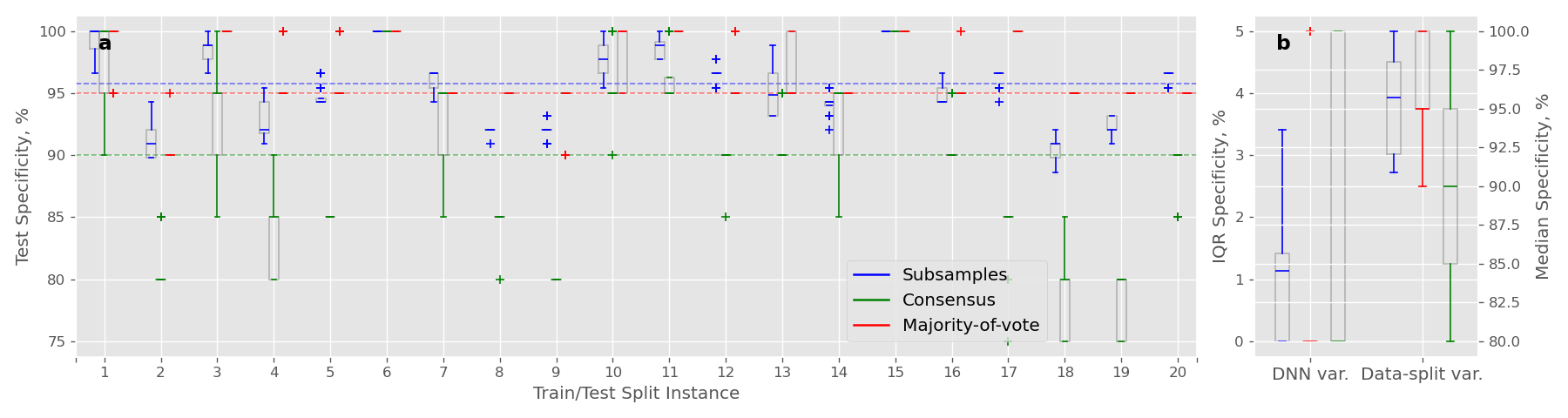
Figure 13S. Test specificity results for the random forest classifier trained with DWT coefficients. For figure details refer to Figure 2S and for the overall median values refer to Table 2.


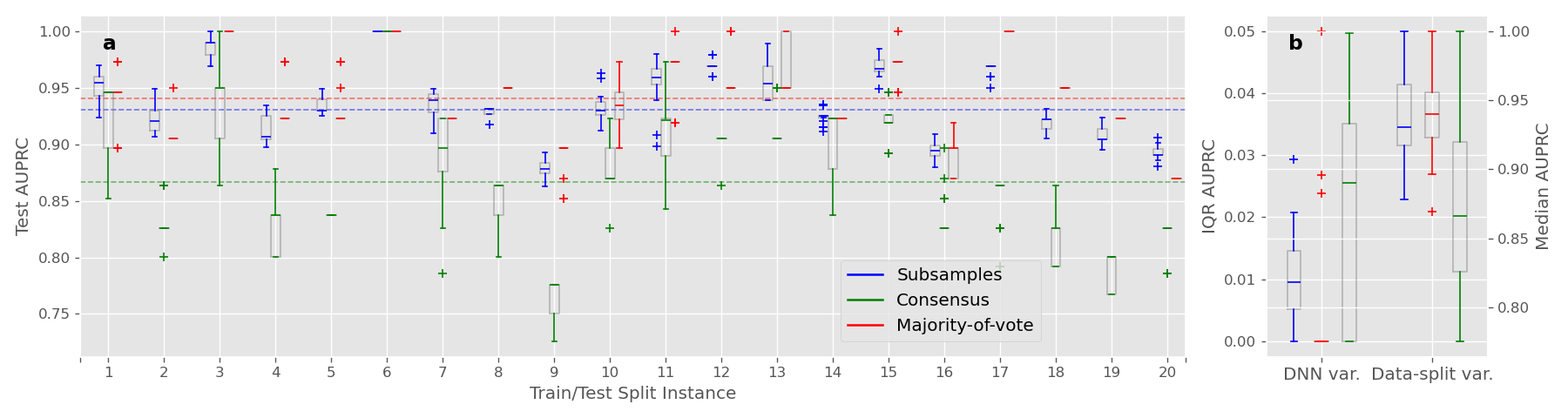
Figure 14S. Test AUPRC results for the random forest classifier trained with DWT coefficients. For figure details refer to Figure 2S and for the overall median values refer to Table 2.


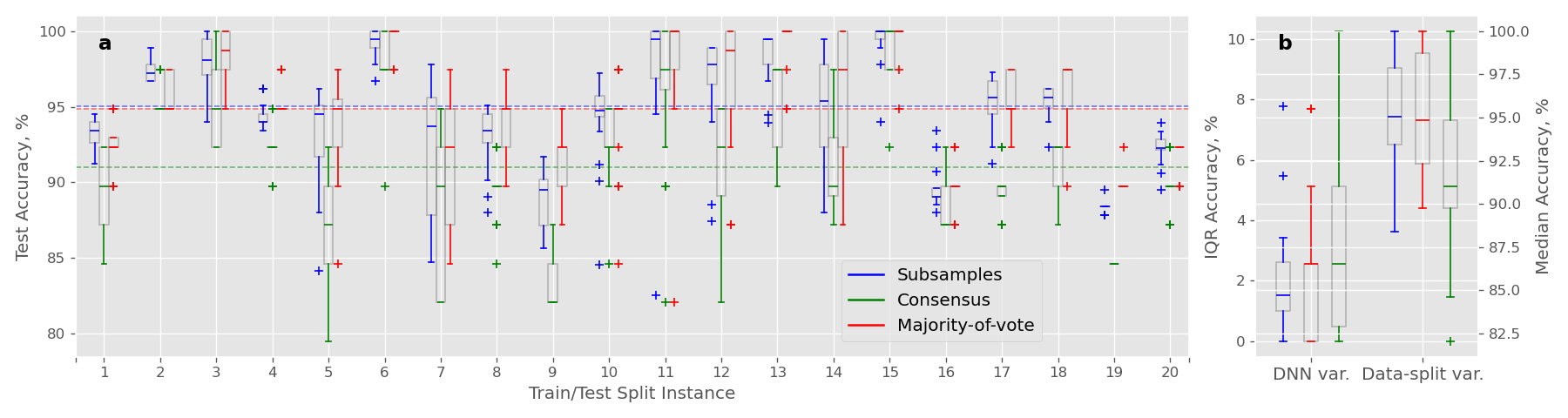
Figure 15S. Test accuracy results for deep neural network trained with PMF profiles. For figure details refer to Figure 2S and for the overall median values refer to Table 2.


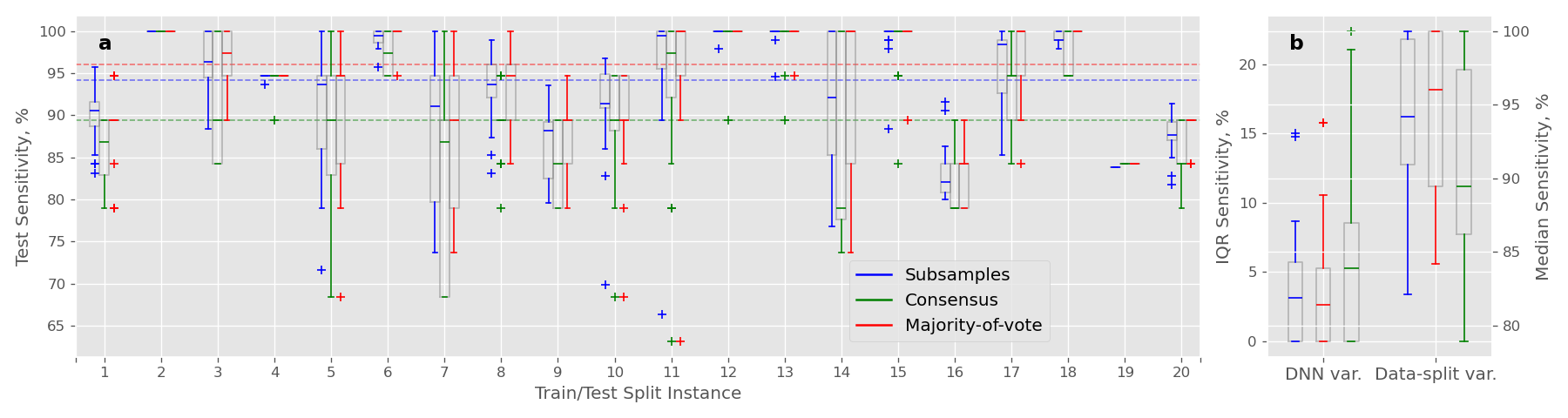
Figure 16S. Test sensitivity results for deep neural network trained with PMF profiles. For figure details refer to Figure 2S and for the overall median values refer to Table 2.


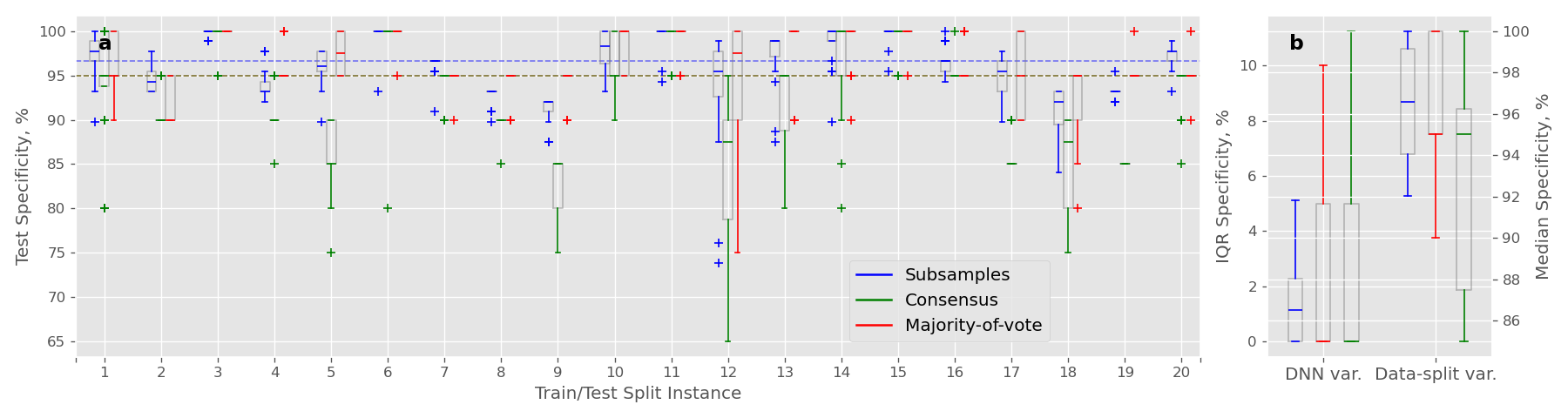
Figure 17S. Test specificity results for deep neural network trained with PMF profiles. For figure details refer to Figure 2S and for the overall median values refer to Table 2.


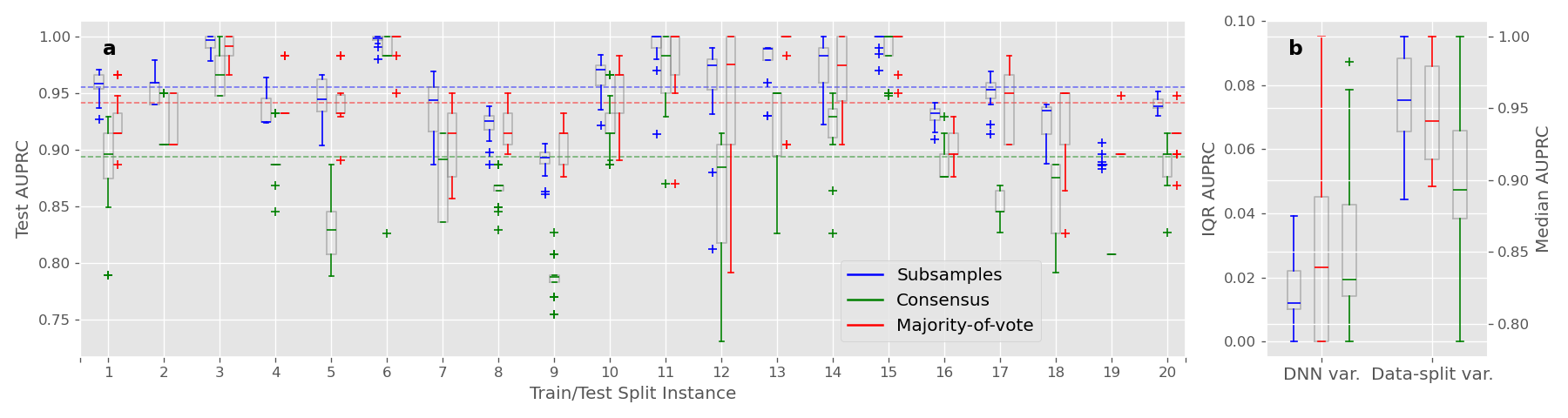
Figure 18S. Test AUPRC results for deep neural network trained with PMF profiles. For figure details refer to Figure 2S and for the overall median values refer to Table 2.


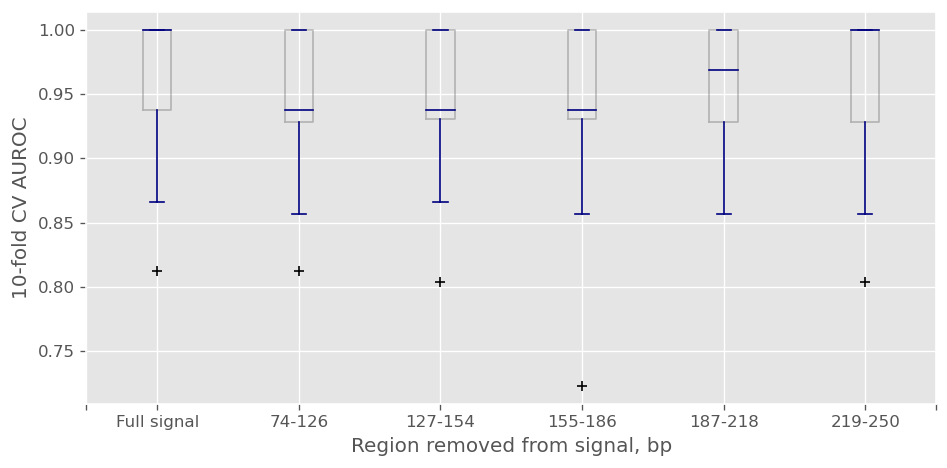
Figure 19S. AUROC boxplots over 10 CV folds for the deep neural network trained with DWT coefficients computed from full PMF signal and from PMF signal with one of 5 regions (left oscillatory region, 74—126 bp; right oscillatory region, 127—154 bp; mode region, 155—186 bp; slope region, 187—218 bp; and tail region, 219—250 bp) zeroed out.
